# Clinical characteristics and outcomes of children with measles requiring pediatric intensive care: a multicenter study in Istanbul

**DOI:** 10.1007/s00431-026-07033-y

**Published:** 2026-05-07

**Authors:** Nihal Akçay, Demet Tosun, İlyas Bingöl, Mehmet Emin Menentoğlu, Esra Şevketoğlu, Fatih Varol, Gözde Alara Kurtiş, Murat Kanğın, Süleyman Bayraktar, Mehmet Arda Kılınç, Emrullah Aygüler, Ufuk Yükselmiş, Ülkem Koçoğlu Barlas, Abdulrahman Özel, Burcu Bursal, Servet Yüce

**Affiliations:** 1 Department of Pediatric Intensive Care Unit, University of Health Sciences Kanuni Sultan Suleyman Training and Research Hospital, Istanbul, Türkiye; 2 Department of Pediatric Intensive Care Unit, University of Health Sciences Bakirköy Dr. Sadi Konuk Training and Research Hospital, Istanbul, Türkiye; 3 Department of Pediatric Intensive Care Unit, University of Health Sciences Sancaktepe Training and Research Hospital, Istanbul, Türkiye; 4 Department of Pediatric Intensive Care Unit, Istanbul Medipol University, Istanbul, Türkiye; 5 Department of Pediatric Intensive Care Unit, University of Health Sciences Haseki Training and Research Hospital, Istanbul, Türkiye; 6 Department of Pediatric Intensive Care Unit, University of Health Sciences Başakşehir Çam Ve Sakura City Hospital, Istanbul, Türkiye; 7 Department of Pediatric Intensive Care Unit, Faculty of Medicine, Istanbul University, Istanbul, Türkiye; 8 Department of Pediatric Intensive Care Unit, University of Health Sciences Kartal Dr. Lütfi Kırdar City Hospital, Istanbul, Türkiye; 9 Department of Pediatric Intensive Care Unit, Istanbul Medeniyet University, Göztepe Prof. Dr. Süleyman Yalçın City Hospital, Istanbul, Türkiye; 10 Department of Pediatric Intensive Care Unit, University of Health Sciences Bağcılar Training and Research Hospital, Istanbul, Türkiye; 11 Department of Public Health, Faculty of Medicine, Istanbul University, Istanbul, 34093 Türkiye

**Keywords:** Measles, Pediatric Intensive Care Unit (PICU), Vaccination, Clinical outcomes, Pediatric infectious diseases

## Abstract

Measles remains a major public health concern despite the availability of effective vaccines. Recent global resurgences, including in Türkiye, have been partly attributed to disruptions in routine immunization and declining vaccination coverage during the COVID-19 pandemic. Data on children with measles requiring pediatric intensive care are limited. We aimed to describe the clinical characteristics and outcomes of children with measles admitted to pediatric intensive care units (PICUs) in a large metropolitan area. This multicenter retrospective study included children < 18 years with laboratory-confirmed measles admitted to ten PICUs in Istanbul, Türkiye, between January and December 2023. Demographic data, vaccination status, clinical features, laboratory findings, treatments, and outcomes were collected. Patients were stratified by vaccination status. Among 5,685 PICU admissions, 53 children (median age 1.3 years) had laboratory-confirmed measles. Ten (18.9%) were vaccinated and 43 (81.1%) were unvaccinated. The median rash duration was longer in vaccinated children (3 vs. 2 days; *p* = 0.027). Rash onset most frequently involved the face in vaccinated children (70%), whereas trunk onset predominated in unvaccinated children (51.2%) (*p* < 0.001). Unvaccinated children were admitted closer to rash onset, indicating a more rapidly evolving clinical course. Rhinorrhea was more common in unvaccinated children (69.8% vs. 20%; *p* = 0.009). Median oxygen saturation at PICU admission was lower in unvaccinated patients (91% vs. 95%; *p* = 0.015). Although ARDS, inotropic support, and invasive ventilation were more frequent in unvaccinated children, these differences were not statistically significant. In multivariable analysis, abnormal chest radiographic findings showed a trend toward association with respiratory support, although this did not reach statistical significance. Overall mortality was 2.3% (1/53), occurring in an unvaccinated infant. *Conclusion*: Measles continues to cause critical illness in young children requiring PICU care. Unvaccinated children tended to present with lower oxygen saturation at admission, suggesting more pronounced respiratory involvement. Maintaining high vaccination coverage remains essential to reduce severe measles-related morbidity.

**Table:** 

**What is Known:** • *Measles outbreaks persist in areas with suboptimal vaccination coverage, with unvaccinated children at higher risk of severe complications.* • *Data on critically ill children with measles, particularly those requiring intensive care, remain limited.*
**What is New:** • *This multicenter study provides contemporary PICU-based data from a recent measles outbreak in a large European metropolitan area, where most critically ill children were unvaccinated and very young.* • *Unvaccinated children showed trends toward lower oxygen saturation and more pronounced respiratory involvement, though findings should be interpreted cautiously due to the small sample size.*

**Supplementary Information:**

The online version contains supplementary material available at 10.1007/s00431-026-07033-y.

## Introduction

Measles is an acute, highly contagious viral disease caused by a paramyxovirus of the genus *Morbillivirus*. Despite the availability of safe and effective vaccines, measles remains an important cause of morbidity and mortality worldwide. The clinical course is typically characterized by fever, malaise, cough, coryza, conjunctivitis, and a maculopapular rash. Most children recover without sequelae within one week; however, severe complications may occur and account for the majority of measles-related deaths. These complications include secondary bacterial infections due to measles-induced immunosuppression, diarrhea, keratoconjunctivitis—which may result in blindness, particularly in vitamin A–deficient populations—otitis media, and pneumonia, the leading cause of measles-associated mortality [1, 2].

In recent years, the global epidemiology of measles has shifted, with a resurgence of cases reported in regions where the disease had previously been well controlled. The World Health Organization (WHO) has documented increasing measles incidence in the European, Eastern Mediterranean, and American regions [1]. Disruptions to routine immunization services during the coronavirus disease 2019 (COVID-19) pandemic contributed substantially to this resurgence. Although temporary declines in measles transmission were observed in 2020 due to non-pharmaceutical public health measures, these gains were offset by reductions in vaccine delivery. It is estimated that approximately 61 million doses of measles-containing vaccines were postponed or missed during the pandemic, leaving large cohorts of susceptible children and creating conditions favorable for outbreaks [3, 4].

Achieving and sustaining ≥ 95% coverage with two doses of measles-containing vaccine is essential for herd immunity. However, this target has not been reached in many countries. In Europe, only a limited number of countries have consistently achieved this threshold. In Türkiye, coverage for the first and second doses has been reported at approximately 90% and 80%, respectively, between 2020 and 2022 [5]. The national immunization program in Türkiye includes two routine doses of the measles–mumps–rubella (MMR) vaccine, administered at 12 months and between 4 and 6 years of age, with an additional early dose recommended for infants aged 9–11 months in outbreak settings [6].

While the epidemiology and clinical features of measles at the population level are well described, data on children with severe disease requiring pediatric intensive care unit (PICU) admission remain limited. Most previous studies include heterogeneous patient populations and varying disease severities, providing only limited insight into the clinical trajectory of critically ill children. Contemporary multicenter data from recent outbreaks are particularly scarce.

Therefore, this multicenter study aimed to characterize the clinical course of laboratory-confirmed measles in children admitted to PICUs in Istanbul. The primary objective was to describe the frequency of measles-related PICU admissions, while secondary objectives included evaluation of clinical and laboratory characteristics, treatment approaches, and patient outcomes.

## Methods

### Study population

This multicenter retrospective study was conducted across ten pediatric intensive care units (PICUs) in Istanbul, Türkiye (Supplementary File 1). All children diagnosed with measles and admitted to these PICUs between January 1, 2023, and December 31, 2023, were screened for eligibility.

Suspected measles cases were evaluated using measles-specific immunoglobulin M (IgM), immunoglobulin G (IgG), and polymerase chain reaction (PCR) testing in accordance with routine clinical practice and World Health Organization (WHO) recommendations. Blood, urine, and nasopharyngeal swab samples were collected from all suspected cases and analyzed at the Public Health Laboratory of the Republic of Türkiye. All patients included in the study had laboratory-confirmed measles and were PCR-positive for measles virus. Measles-specific IgM was positive in 75.5% (40/53) of patients; although reviewed as part of the diagnostic workup, IgM positivity was not used as a standalone inclusion criterion. According to WHO definitions, a confirmed measles case requires laboratory evidence such as detection of measles-specific IgM antibodies, isolation of measles virus, identification of viral RNA by PCR, or a fourfold or greater rise in measles-specific IgG titers in paired serum samples [7].

Children younger than 18 years with laboratory-confirmed measles who required PICU admission during the study period were eligible. Laboratory confirmation was based on PCR testing and/or measles-specific serology as part of routine diagnostic evaluation. Patients with clinically suspected measles but negative PCR and measles-specific IgM results were excluded.

PICU admission was based on the presence of one or more of the following indications: respiratory failure, hemodynamic instability, shock, multi-organ dysfunction syndrome (MODS), or altered level of consciousness requiring intensive monitoring and supportive care.

### Immunization status

According to the national immunization schedule in Türkiye, the measles–mumps–rubella (MMR) vaccine is routinely administered in two doses. Prior to July 1, 2020, these were scheduled at 12 months and 6 years of age. Following updates to the national immunization program, the two routine doses have been administered at 12 and 48 months of age. In addition, an extra MMR dose may be administered at 9 months of age in regions with increased outbreak risk.

Vaccination status was verified using official vaccination cards issued by the Ministry of Health and/or the national electronic health record system (e-Nabız). When official documentation was unavailable, vaccination history was recorded based on parental or caregiver report.

Measles vaccination status was categorized as:

- **Unvaccinated:** no documented measles-containing vaccine dose
- **Partially vaccinated:** receipt of a single MMR dose or incomplete vaccination according to age-specific national recommendations
- **Fully vaccinated:** receipt of two or more MMR doses consistent with the national schedule

Vaccines administered less than 14 days before symptom onset were considered unlikely to confer adequate protective immunity and were classified accordingly. Reasons for incomplete or absent vaccination were recorded only when explicitly documented in medical records.

For inferential analyses, patients who had received at least one MMR dose (partially or fully vaccinated) were combined into a single group due to the limited sample size and to improve statistical robustness.

### Data collection

Data were retrospectively extracted from medical records at each participating center using standardized case report forms. Collected variables included demographics, gestational age, comorbidities, immunization status, clinical presentation, date of symptom onset, Pediatric Risk of Mortality III (PRISM III) and Pediatric Logistic Organ Dysfunction-2 (PELOD-2) scores, admission diagnosis, duration of illness, vital signs at admission (heart rate, body temperature, and peripheral oxygen saturation [SpO₂]), laboratory findings, concurrent infections, complications, need for respiratory support (noninvasive or invasive), therapeutic interventions, length of hospital stay (LOS), and mortality.

PRISM III scores were calculated using the most abnormal clinical and laboratory values recorded within the first 24 h of PICU admission. PELOD-2 scores were calculated based on data obtained at the time of PICU admission. Admission laboratory parameters and vital signs were recorded at PICU entry. Respiratory support modalities were documented if administered at any point during PICU stay. LOS and mortality outcomes were recorded until hospital discharge or death.

The severity of pediatric acute respiratory distress syndrome (pARDS) was classified as mild, moderate, or severe according to the Second Pediatric Acute Lung Injury Consensus Conference criteria [8]. All patients underwent echocardiographic evaluation by a pediatric cardiologist. To ensure standardization, reported vital signs and laboratory values represent the first measurements obtained at PICU admission.

### Definition of co-infection

Co-infection was defined based on a comprehensive assessment incorporating clinical, laboratory, and radiological findings. Detection of viral or bacterial pathogens on multiplex respiratory PCR panels alone was not considered sufficient evidence of causality. Given that measles itself can cause primary viral pneumonia, co-infection was diagnosed when radiological involvement was disproportionate to typical measles pneumonia patterns and supported by compatible clinical findings and laboratory indicators suggestive of an additional infectious process.

### Definition of loss of appetite

Loss of appetite was defined based on caregiver report of reduced oral intake compared to baseline prior to PICU admission, thereby reflecting the pre-admission clinical course rather than feeding limitations related to PICU interventions.

### Statistical analysis

Statistical analyses were performed using IBM SPSS Statistics for Windows, Version 29.0 (IBM Corp., Armonk, NY, USA). Continuous variables were reported as medians with interquartile ranges (IQR), and categorical variables as frequencies and percentages. Comparisons between vaccinated and unvaccinated groups were conducted using Fisher’s exact test, Yates-corrected chi-square test, or Monte Carlo simulations for categorical variables, as appropriate. The Mann–Whitney U test was used for continuous variables.

A multivariable logistic regression model was constructed to identify factors associated with the need for respiratory support. Age was included as a continuous variable. Vaccination status (≥ 1 MMR dose vs. unvaccinated), comorbidity, co-infection, and abnormal chest radiographic findings were included based on clinical relevance. Given the limited sample size, the model was kept parsimonious to reduce the risk of overfitting. Variables included in the multivariable model were selected based on clinical relevance and prior literature, rather than automated selection procedures. No formal variable selection procedures (e.g., stepwise methods) were applied.

A two-sided *p*-value < 0.05 was considered statistically significant.

### Handling of missing data

Given the retrospective design, some variables were missing in a limited number of cases. No data imputation was performed. Analyses were conducted using available-case (complete-case) analysis for each variable. The number of observations included in each analysis was reported where relevant. The proportion of missing data was < 2% for all variables. Given the low rate of missingness, no data imputation was performed, and analyses were conducted using complete-case data.

### Ethical considerations

The study was approved by the Ethics Committee of the Health Sciences University, Kanuni Sultan Süleyman Training and Research Hospital (KAEK/2024.01.19). Institutional review board approval was also obtained from each participating center in accordance with national and local regulations. Due to the retrospective nature of the study, the requirement for informed consent was waived. The study was conducted in accordance with the Declaration of Helsinki.

## Results

Between January and December 2023, a total of 5,685 children were admitted to ten PICUs in Istanbul. Among these, 53 children with laboratory-confirmed measles were included in the analysis.

**Fig. 1:**
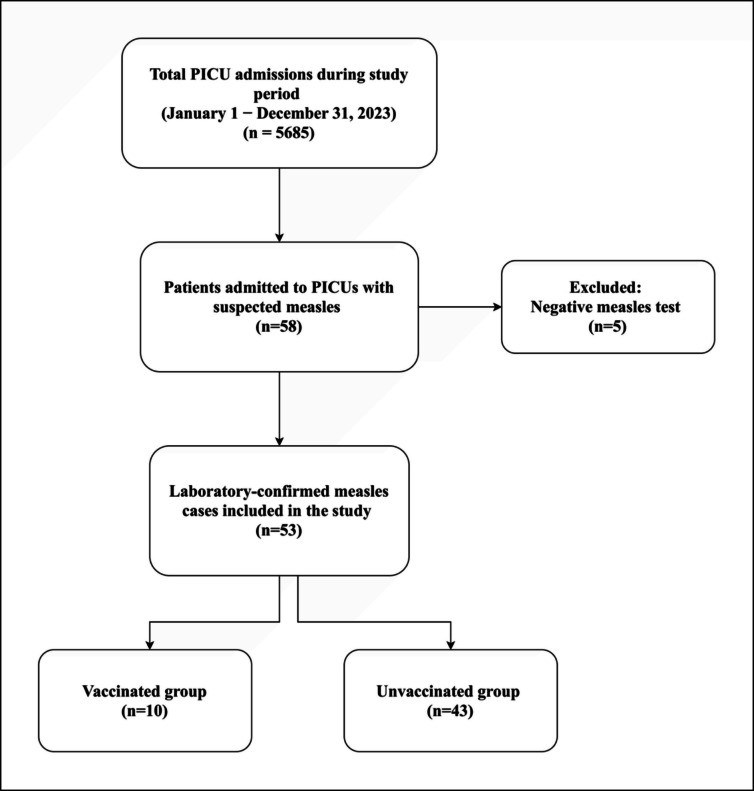
Flow diagram of patient selection and study inclusion

Of the cohort, 10 (18.9%) had received at least one dose of MMR vaccine, whereas 43 (81.1%) had not received any or had received only a single dose and were therefore grouped as unvaccinated for analytical purposes (Fig. 1). For descriptive purposes, vaccination status was further categorized as unvaccinated, partially vaccinated, and fully vaccinated. Comparative data on clinical characteristics and outcomes according to vaccination status are summarized in Tables 1 and 2**.**

**Table 1:** Baseline clinical characteristics of children with measles stratified by vaccination status

Characteristics	Vaccinated group (n = 10)	Non-vaccinated group (n = 43)	All patients (n = 53)	*p*-values
**Age, years, median (IQR)**	2.1 (1.1–3.4)	1.1 (0.5–2.8)	1.3 (0.6–2.8)	*0.149*^*1*^
**Ethnicity**				*0.058*^*2*^
Turks	90% (9/10)	46.5% (20/43)	54.7% (29/53)	
Others*	10% (1/10)	53.4% (23/43)	45.3% (24/53)	
**Female gender, n (%)**	50% (5/10)	51.2% (22/43)	50.9% (27/53)	*0.611*^*2*^
**Gestational age, median (IQR)**	38 (36–40)	38 (37–40)	38 (37–40)	*0.805*^*1*^
**Birth weight, g, median (IQR)**	3500 (3400–3950)	3300 (2900–3500)	3300 (2990–3500)	*0.160*^*1*^
**Comorbidities, n (%)**	10% (1/10)	20.9% (9/43)	18.9% (10/53)	*0.827*^*1*^
**Duration of measles-related maculopapular rash in admission, median (IQR)**	3 (2–7)	2 (1–4)	2 (1–4)	***0.027***^*1*^
**The onset area of the maculopapular rash**				** < *0.001***^*2*^
Facial and cranial regions	70% (7/10)	44.2% (19/43)	49.1% (26/53)	
Trunk	0% (0/10)	51.2% (22/43)	41.5% (22/53)	
Cervical and dorsal regions	30% (3/10)	0% (0/43)	5.7% (3/53)	
**Koplik spots**	10% (1/10)	4.7% (2/43)	5.7% (3/53)	*0.473*^*2*^
**Exposure to cigarette smoke, n (%)**	50% (5/10)	30.2% (13/43)	34% (18/53)	*0.279*^*2*^
**Household contacts history, n (%)**	50% (5/10)	58.1% (25/43)	56.6% (30/53)	*0.730*^*2*^
**Coinfections, n (%)**	0% (0/10)	25.6% (11/43)	20.8% (11/53)	*0.098*^*2*^
**Symptoms, n (%)**				
Loss of appetite	90% (9/10)	90.6% (39/43)	90.6% (48/53)	*1.000*^*2*^
Rash	100% (10/10)	81.1% (37/43)	88.7% (47/53)	*0.581*^*2*^
Fever	100% (10/10)	97.7% (42/43)	98.1% (52/53)	*0.581*^*2*^
Respiratory distress	90% (9/10)	83.7% (36/43)	84.9% (45/53)	*0.527*^*2*^
Pneumonia	%100 (10/10)	79.1% (34/43)	83% (44/53)	*0.127*^*2*^
Cough	80% (8/10)	81.4% (35/43)	81.1% (43/53)	*0.612*^*2*^
Rhinorrhea	20% (2/10)	69.8% (30/43)	60.4% (32/53)	***0.009***^*2*^
Lymphadenopathy	30% (3/10)	55.8% (24/43)	50.9% (27/53)	*0.175*^*2*^
Conjunctivitis	40% (4/10)	32.6% (14/43)	34% (18/53)	*0.719*^*2*^
Sore throat	40% (4/10)	25.6% (11/43)	28.3% (15/53)	*0.442*^*2*^
Diarrhea	30% (3/10)	11.6% (5/43)	15.1% (8/53)	*0.163*^*2*^
Arthritis	10% (1/10)	9.3% (4/43)	9.4% (5/53)	*0.665*^*2*^
Otitis	0% (0/10)	7.0% (3/43)	5.7% (3/53)	*0.527*^*2*^
Myocarditis/Pericarditis	20% (2/10)	0% (0/43)	3.8% (2/53)	***0.033***^*2*^
Encephalitis	0% (0/10)	4.7% (2/43)	3.8% (2/53)	*1.000*^*2*^
**PRISM-3, median (IQR)**	4 (2–9)	4 (3–7)	4 (3–7)	*0.599*^*1*^
**PELOD-2, median (IQR)**	3 (3–4)	3 (3–6)	3 (3–6)	*0.957*^*1*^
**Vital and Clinical signs**				
Heart rate (beats/min), median (IQR)	136 (130–150)	135 (123–148)	135 (125–148)	*0. 918*^*1*^
Respiratory rate (breaths/min), median (IQR)	37 (30–45)	40 (31–45)	40 (31–45)	*0.591*^*1*^
SBP (mm Hg), median (IQR)	105 (80–112)	90 (85–100)	91 (85–104)	*0.459*^*1*^
DBP (mm Hg), median (IQR)	60 (50–61)	55 (50–65)	57 (50–62)	*0.757*^*1*^
SpO_2_ (%), median (IQR)	95 (93–96)	91 (88–96)	92 (90–96)	***0.015***^*1*^

* “Others” includes patients of non-Turkish nationality (e.g., refugees and immigrants)

Data are presented as median (IQR) or n (%), unless otherwise specified. Comparisons were performed using the ^1^Mann–Whitney U test for continuous variables and the ^2^chi-square or Fisher’s exact test for categorical variables. Percentages may not total 100% due to rounding.

Abbreviations: *IQR* interquartile range, *SBP* systolic blood pressure, *DBP* diastolic blood pressure, *SpO*_*2*_ peripheral oxygen saturation, *ALT* alanine aminotransferase, *AST* aspartate aminotransferase, *APTT* activated partial thromboplastin time, *CRP* C-reactive protein, *PICU* pediatric intensive care unit, *ARDS* acute respiratory distress syndrome**.**

**Table 2:** Laboratory findings and clinical interventions in children with measles stratified by vaccination status

Characteristics	Vaccinated group (*n* = 10)	Non-vaccinated group (*n* = 43)	All patients (*n* = 53)	*p*-values
**Laboratory values**				
White blood cell count (10^3^/µL), median (IQR)	8165 (5500–13550)	8000 (5070–11900)	8000 (5360–12050)	*0.481*^*1*^
Lymphocyte count, (10^3^/µL), median (IQR)	2760 (1580–3700)	2490 (1380–4360)	2520 (1380–4160)	*0.883*^*1*^
Neutrophil count, (10^3^/µL), median (IQR)	4955 (3600–6670)	4030 (2190–5730)	4410 (2460–5730)	*0.184*^*1*^
Platelet count, (10^3^/µL), median (IQR)	241500 (103000–374000)	301000 (214000–444000)	301000 (202000–438000)	*0.290*^*1*^
Hemoglobin, (g/dL), median (IQR)	10.2 (9–11.5)	9.9 (9.1–11.3)	9.9 (9.1–11.3)	*0.937*^*1*^
Hematocrit (%), median (IQR)	30.3 (27–37.2)	32.6 (29.4–35.3)	32.3 (29.3–35.3)	*0.413*^*1*^
ALT (U/L), median (IQR)	51.6 (36–63)	44 (35.3–63)	44.4 (35.6–63)	***0.017***^*1*^
AST (U/L), median (IQR)	30.5 (22–38)	17 (14.1–29.3)	19.4 (14.1–33)	*0.394*^*1*^
Urea (mg/dL), median (IQR)	18.6 (12.8–26.4)	15.4 (10–19.6)	15.4 (11.4–20.1)	*0.211*^*1*^
Creatinine (mg/dL), median (IQR)	0.37 (0.24–0.44)	0.21 (0.17–0.25)	0.23 (0.17–0.32)	***0.008***^*1*^
Sodium (mEq/L), median (IQR)	138 (135–139)	135 (133–138)	135 (134–138)	*0.209*^*1*^
Potassium (mEq/L), median (IQR)	4.4 (3.9–4.9)	4.3 (4–4.7)	4.3 (4–4.8)	*0.964*^*1*^
Calcium (mg/dL), median (IQR)	9 (8.4–9.8)	8.8 (8.6–9.1)	8.9 (8.5–9.1)	*0.255*^*1*^
Creatine kinase (U/L), median (IQR)	80 (62–155)	104 (70–192)	102 (70–171)	*0.510*^*1*^
Lactate dehydrogenase (U/L), median (IQR)	655 (380–799)	539 (393–685)	543 (393–713)	*0.525*^*1*^
C-reactive protein (mg/L), median (IQR)	23.8 (20.4–47.4)	10 (3.1–44)	14 (4.3–44)	***0.048***^*1*^
Procalcitonin (ng/mL), median (IQR)	0.7 (0.47–2.6)	0.28 (0.11–1.4)	0.39 (0.14–1.4)	***0.037***^*1*^
Ferritin (ng/mL), median (IQR)	688.4 (298–837)	249 (140–397)	270 (141–415)	*0.052*^*1*^
Fibrinogen (mg/dL), median (IQR)	290 (182–527)	269 (245–400)	269 (245–400)	*0.980*^*1*^
Prothrombin time (s), median (IQR)	12.3 (12.1–16)	14 (12.1–14.8)	13.7 (12.1–14.9)	*0.698*^*1*^
APTT (s), median (IQR)	29 (18.1–33.4)	27.5 (24.2–32.2)	27.7 (24.2–32.2)	*0.817*^*1*^
INR, median (IQR)	1 (0.92–1.2)	1.1 (1–1.1)	1.1 (0.99–1.1)	*0.585*^*1*^
Pro-BNP (pg/Ml), median (IQR)	99 (76.1–459)	365 (160–1860)	360 (156–1680)	*0.150*^*1*^
**Baseline Blood Gas Values**				
pH, median (IQR)	7.37 (7.36–7.4)	7.37 (7.3–7.42)	7.37 (7.31–7.42)	*0.407*^*1*^
pCO_2_ (mmHg), median (IQR)	38 (36–41)	39.8 (34–45.7)	39 (34–44)	*0.394*^*1*^
HCO3 (mmHg), median (IQR)	23 (21.3–23.2)	23 (20–24.3)	23 (20.4–24.3)	*0.941*^*1*^
BE (mmol/L), median (IQR)	−2.15 (−3.5–−0.55)	−3.05 (−4.15–0.35)	−2.95 (−4–0.1)	*0.580*^*1*^
Lactate (mmol/L), median (IQR)	1.92 (1.35–2.55)	1.9 (1.38–2.8)	1.9 (1.38–2.6)	*0.832*^*1*^
**Chest X-ray, n (%)**				*0.551*^*2*^
İnterstitial	40% (4/10)	23.2% (10/43)	26.4% (14/53)	
Lobar	30% (3/10)	46.4% (20/43)	43.3% (23/53)	
Hilar fullness	0% (0/10)	9.3% (4/43)	7.5% (4/53)	
Nonspesific changes	30% (3/10)	0% (0/43)	5.7% (3/53)	
Subcutaneous emphysema	0% (0/10)	2.3% (1/43)	1.8% (1/53)	
**Maximum respiratory support, n (%)**				*0.833*^*2*^
Continuous oxygen therapy	30% (3/10)	23.2% (10/43)	24.5% (13/53)	
High-flow nasal cannula oxygen therapy (HFNC)	40% (4/10)	41.8% (18/43)	41.5% (22/53)	
Noninvasive ventilation (NIV)	20% (2/10)	13.9% (6/43)	15% (8/53)	
Invasive mechanical ventilation (IMV)	10% (1/10)	20.9% (9/43)	18.8% (10/53)	
**pARDS n (%)**				*0.820*^*2*^
Mild	40% (4/10)	46.4% (20/43)	45.3% (24/53)	
Moderate	20% (2/10)	13.9% (6/43)	15.1% (8/53)	
Severe	10% (1/10)	18.6% (8/43)	16.9% (9/53)	
**Inotrope Treatment n (%)**	0% (0)	11.6% (5/43)	9.4% (5/53)	*0.570*^*2*^
**Lengths of HFNC, median (IQR), [range]**	5 (4–7) [2—7]	3 (2–4) [1—6]	3 (2–5) [1—7]	***0.035***^*1*^
**Lengths of Noninvasive Mechanical Ventilation, median (IQR), [range]**	4 (3–5) [3—5]	4 (3–6) [2—8]	4 (3–6) [2—8]	*0.912*^*1*^
**Lengths of Invasive Mechanical Ventilation, median (IQR) [range]**	8 (8–8) [1—17]	8 (5–16) [5—21]	8 (5–16) [1—21]	*1.000*^*1*^
**Lengths of hospital stay, median (IQR), [range]**	12.5 (8–18) [3—79]	10 (7–14) [2—40]	11 (7–14) [2—79]	*0.657*^*1*^
**Lengths of PICU stay, median (IQR), [range]**	6 (4–13) [1—23]	6 (3–9) [2—32]	6 (3–9) [1—32]	*0.697*^*1*^
**Mortality, n (%)**	0% (0/10)	2.3% (1/43)	1.9% (1/53)	*1.000*^*2*^

Data are presented as median (IQR) or n (%), unless otherwise specified. Comparisons were performed using the ^1^Mann–Whitney U test for continuous variables and the ^2^chi-square or Fisher’s exact test for categorical variables. Percentages may not total 100% due to rounding.

**Abbreviations:** *IQR* interquartile range, *ALT* alanine aminotransferase, *AST* aspartate aminotransferase, *APTT* activated partial thromboplastin time, *CRP* C-reactive protein, *PICU* pediatric intensive care unit, *ARDS* acute respiratory distress syndrome, *HFNC* high-flow nasal cannula, *NIV* noninvasive ventilation, *IMV* invasive mechanical ventilation, *BE* base excess, *pCO*_*2*_ partial pressure of carbon dioxide.

The overall median age was 1.3 years (IQR 0.6–2.8), with no age difference between vaccinated and unvaccinated children (*p* = 0.149). A borderline difference was observed in ethnic background, with a higher proportion of Turkish nationality in the vaccinated group (90% vs. 46.5%, *p* = 0.058). Sex, gestational age, birth weight, and presence of comorbidities were comparable between groups.

A measles-related maculopapular rash was present in 96.2% (51/53) of patients. The median rash duration was longer in vaccinated children (3 vs. 2 days, *p* = 0.027). Rash onset most commonly involved the facial and cranial regions in vaccinated children (70%), whereas trunk onset predominated in unvaccinated children (51.2%, *p* < 0.001). The two patients without rash underwent PCR testing because they were close contacts residing in a social care institution with concurrent confirmed measles cases.

**Fig. 2:**
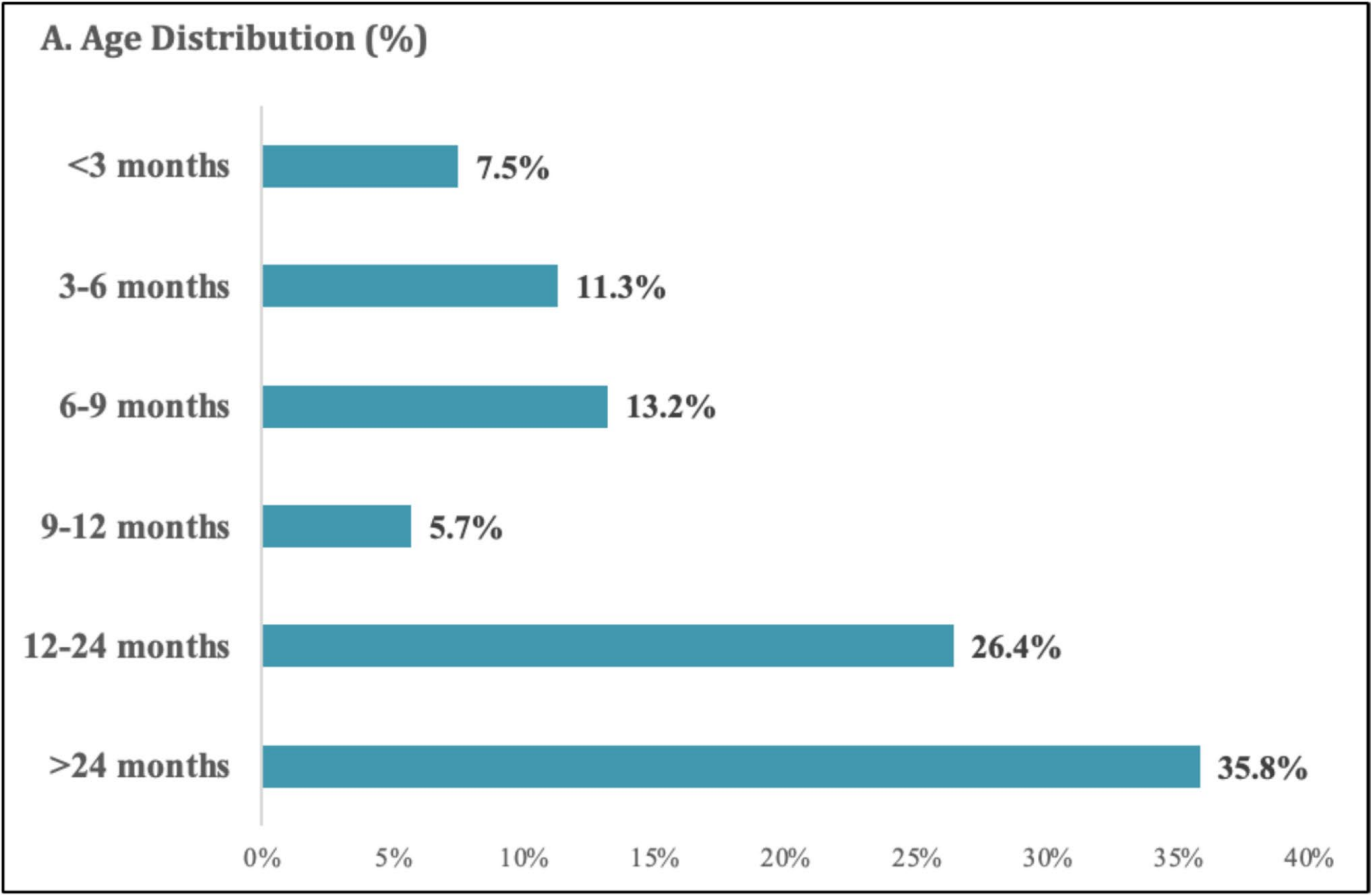

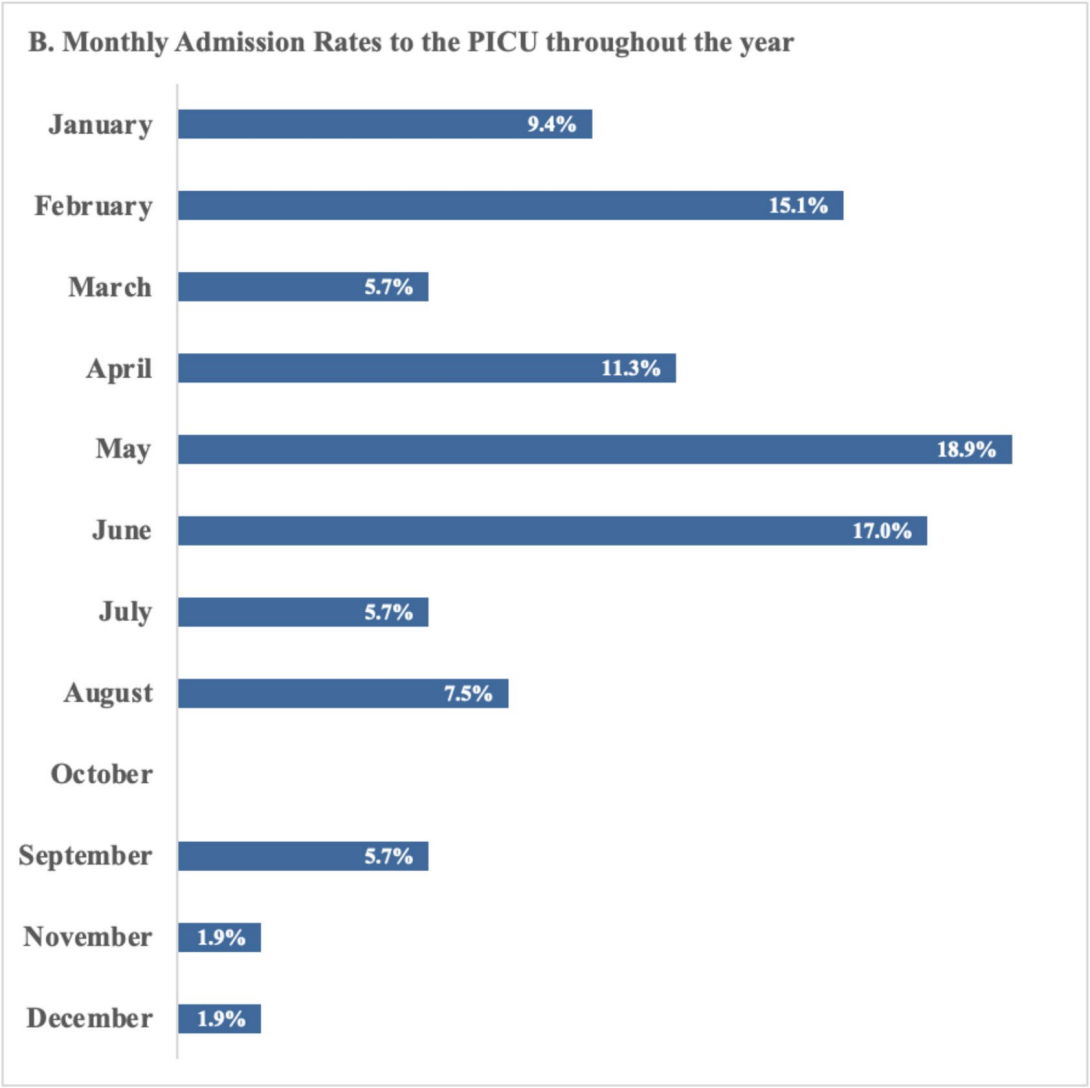

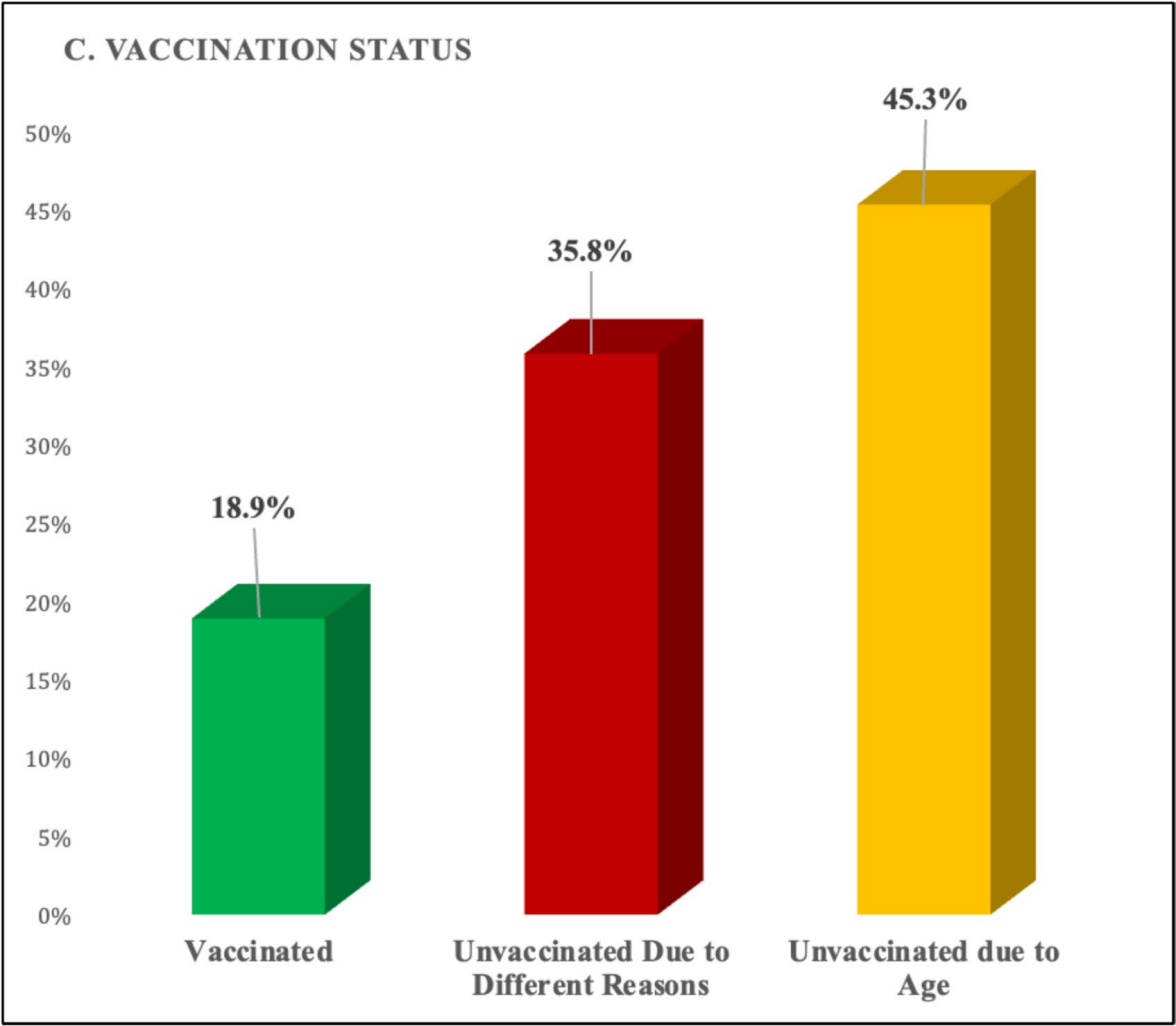
Demographic and epidemiologic distribution of measles cases requiring pediatric intensive care. (**A**) Age distribution of children with laboratory-confirmed measles admitted to pediatric intensive care units (PICUs). (**B**) Monthly distribution of measles-related PICU admissions during the 2023 study period. (**C**) Vaccination status of included patients. Among unvaccinated children, a proportion were ineligible due to age, while others were unvaccinated for various reasons. Footnotes: • Values are percentages unless otherwise indicated. • Data were derived from medical records and documented vaccination histories. • PCR positivity was the primary diagnostic criterion; measles IgM testing was available in a subset. • Percentages are rounded to one decimal place

Figure 2A shows the age distribution, Fig. 2B the monthly admission pattern, and Fig. 2C the vaccination status. Among unvaccinated children, 35.8% were unvaccinated due to undocumented immigration status, immunodeficiency, or parental refusal, whereas 45.3% were below the eligible vaccination age.

Clinical manifestations (including fever, rash, anorexia, cough, and respiratory distress) were similarly distributed between groups. Rhinorrhea was significantly more frequent in unvaccinated children (69.8% vs. 20%, *p* = 0.009).

Co-infections were identified only in unvaccinated patients (11/43, 25.6%), although the difference did not reach statistical significance (*p* = 0.098). Detected pathogens included *Haemophilus influenzae, Streptococcus pneumoniae, cytomegalovirus (CMV), influenza A,* and *SARS-CoV-2*. Among patients with *S. pneumoniae* (*n* = 3) and *H. influenzae* (*n* = 2), chest imaging demonstrated lobar consolidation and focal findings consistent with bacterial pneumonia. Two patients had CMV detected by PCR. These patients demonstrated bilateral ground-glass opacities and diffuse interstitial involvement on imaging; however, the clinical significance of CMV detection in this context remains uncertain. However, the detection of bacterial pathogens from respiratory samples may represent colonization rather than true infection, particularly in the absence of microbiological confirmation from sterile sites.

PRISM III and PELOD-2 scores did not differ between groups. Median oxygen saturation at admission was lower in unvaccinated children (91% [IQR 88–96] vs. 95% [IQR 93–96], *p* = 0.015). Other vital signs were comparable.

CRP levels were higher in vaccinated children, whereas other laboratory parameters were similar. No group differences were observed in the type or duration of respiratory support, PICU stay, or total hospital stay.

Overall, 40 children (75.5%) required respiratory support. The remaining patients were admitted for intensive monitoring because of clinical concern at presentation, including risk of respiratory deterioration and/or hemodynamic instability, although respiratory support was not ultimately required. No differences were observed in demographic or clinical characteristics between those who did and did not require support. However, co-infection was more frequent among patients requiring invasive mechanical ventilation (*p* < 0.05).

Chest radiographic findings were similar between groups, with interstitial and lobar infiltrates predominating. Most patients met criteria for mild-to-moderate pARDS, with no difference in severity (*p* = 0.820).

Inotropic support was required in five unvaccinated children (11.6%) and none of the vaccinated children (*p* = 0.570). One death occurred in an unvaccinated infant (2.3%).

### Fatal case

The single fatal case involved a 3-month-old unvaccinated infant who was ineligible for vaccination due to age. She presented with fever, poor feeding, conjunctivitis, cough, and rash. On admission, she exhibited severe respiratory distress and hemodynamic instability. Despite invasive mechanical ventilation and inotropic support, she developed severe pARDS and multi-organ failure and died on PICU day 32.

### Multivariable analysis

A multivariable logistic regression model was constructed to evaluate factors associated with the need for respiratory support. In this analysis, abnormal chest radiographic findings were associated with a higher likelihood of requiring respiratory support (OR 5.09, 95% CI 0.89–29.27; *p* = 0.067), although this association did not reach statistical significance. Age, vaccination status, comorbidity, and co-infection were not independently associated with the outcome in this model (Table 3).

**Table 3:** Multivariable logistic regression analysis for need for respiratory support

Variable	OR	95% CI	*p*-value
Age (months)	0.996	0.974–1.017	0.687
≥ 1 MMR dose (vs unvaccinated)	1.45	0.22–9.56	0.691
Comorbidity	1.37	0.15–12.15	0.772
Co-infection	3.52	0.25–49.18	0.342
Abnormal chest X-ray	5.09	0.89–29.27	0.067

Odds ratios (ORs) were derived from a multivariable logistic regression model. Age was included as a continuous variable. Vaccination status was coded as ≥ 1 MMR dose vs. unvaccinated. Abnormal chest radiographic findings were defined as interstitial or lobar infiltrates. **Variables included in the model were selected based on clinical relevance.** Given the limited sample size, results should be interpreted with caution.

## Discussion

This multicenter study provides contemporary data on children with laboratory-confirmed measles requiring pediatric intensive care in a large metropolitan setting. Our findings highlight that measles continues to cause critical illness in young children, particularly among those who are unvaccinated or too young to be vaccinated.

The resurgence of measles observed in our cohort aligns with recent epidemiological reports from the European Centre for Disease Prevention and Control, which documented increasing measles activity across Europe following the COVID-19 pandemic [9]. Pandemic-related disruptions to routine immunization have resulted in immunity gaps, particularly in infants and young children. The median age of 1.3 years in our cohort is consistent with global data showing that measles disproportionately affects children under five years of age [1, 10, 11]. This age distribution underscores the vulnerability of infants and toddlers, many of whom are either unvaccinated or have not yet completed the recommended vaccination schedule.

The high proportion of children without complete measles immunization in our cohort reflects broader challenges in achieving optimal vaccination coverage. The observed ethnic and demographic differences between groups may partly explain this pattern, potentially reflecting migration dynamics and disparities in healthcare access. Türkiye hosts a large immigrant population, and culturally sensitive vaccination strategies and targeted outreach programs may therefore be necessary to improve coverage in underserved communities [5, 12].

Clinical presentation in our cohort was generally consistent with classical measles descriptions. However, the relatively frequent trunk-onset rash observed among unvaccinated children represents a deviation from the typical cephalocaudal progression. While this finding should be interpreted cautiously, it may reflect variability in host immune response, timing of clinical evaluation, or differences in viral dynamics [14]. Given the exploratory nature of this observation, further studies are needed before drawing pathophysiological conclusions.

Interpretation of rash characteristics in critically ill children is inherently challenging. In patients requiring intensive care, clinical attention may be primarily directed toward life-threatening conditions such as respiratory or hemodynamic instability, potentially leading to underrecognition or incomplete documentation of dermatological findings. Therefore, the observed differences in rash patterns should be interpreted with caution, as they may partly reflect differences in clinical assessment rather than true biological variation.

Co-infections were identified in approximately one-quarter of unvaccinated patients and were associated with a higher likelihood of invasive respiratory support. This is consistent with the well-documented immunosuppressive effects of measles and its predisposition to secondary bacterial infections, particularly pneumonia [13, 15]. The high proportion of patients requiring respiratory support in our cohort further illustrates the potential severity of measles in critically ill children. It should be noted that the identification of bacterial pathogens such as *Haemophilus influenzae* and *Streptococcus pneumoniae* from respiratory samples may reflect colonization rather than true infection, particularly in the absence of confirmation from sterile sites. Therefore, these findings should be interpreted with caution, and the contribution of bacterial co-infection may have been overestimated in some cases.

The detection of CMV in a small number of patients should be interpreted with caution. In critically ill children, CMV positivity may reflect viral reactivation or incidental detection rather than true pathogenic infection, particularly in the absence of immunosuppression.

Although unvaccinated children tended to present with lower oxygen saturation and showed higher rates of ARDS, inotropic support, and invasive ventilation, these differences did not reach statistical significance. This may reflect limited statistical power rather than absence of clinical relevance. Previous literature consistently reports more severe disease courses among unvaccinated individuals [14].

Our regression analysis suggested that abnormal chest radiographic findings may be associated with an increased likelihood of requiring respiratory support; however, this finding did not reach statistical significance and should be interpreted cautiously.

The mortality rate in our cohort was low, with one death occurring in an unvaccinated infant who was below the eligible vaccination age. While reassuring, this should not obscure the substantial morbidity associated with severe measles. WHO data indicating a high measles burden in Türkiye in 2023 further emphasize the public health relevance of these findings [19].

### Limitations

Several limitations should be acknowledged. The retrospective design may introduce information bias and precludes causal inference. The study population consisted exclusively of PICU patients, representing the most severe end of the clinical spectrum and limiting generalizability. In addition, the absence of a comparator group (e.g., hospitalized measles patients not requiring PICU admission) limits our ability to distinguish factors associated with critical illness from those related to measles infection in general.

The relatively small sample size restricted statistical power and limited the interpretation of subgroup and regression analyses. Vaccination status was simplified for analysis and did not account for timing or completeness of immunization. Some findings—particularly those related to rash characteristics—should be considered hypothesis-generating.

Due to the multicenter retrospective design, center-level clustering could not be accounted for in the regression analysis because consistent center identifiers were not available in the dataset. This may have introduced unmeasured heterogeneity across participating PICUs. In addition, data on the total number of hospitalized measles cases outside the PICU were not consistently available across participating centers, limiting our ability to contextualize the proportion of patients requiring intensive care.

Blood pressure values were recorded as absolute measurements rather than age-adjusted percentiles due to limitations of the retrospective dataset, which may limit interpretation in relation to age-specific norms. Furthermore, PICU admission decisions were based on real-world clinical judgment at each center, and some patients were admitted for close monitoring despite not ultimately requiring respiratory support. Because of the retrospective design, a reliable post hoc reclassification of PICU appropriateness or exclusion of such cases was not feasible.

Finally, long-term outcomes after PICU discharge were not assessed.

## Conclusion

Measles continues to impose a significant burden on pediatric critical care, particularly among unvaccinated or incompletely vaccinated children. Although mortality was low, the high rate of respiratory support and complications highlights the potential severity of measles in this population. Maintaining high vaccination coverage and closing immunity gaps remain essential strategies to prevent severe disease and future outbreaks. Strengthening targeted vaccination efforts in vulnerable populations should remain a public health priority.

## Supplementary Information

Below is the link to the electronic supplementary material.

Supplementary file1 (DOCX 14 KB)

## Data Availability

De-identified data are available from the corresponding author upon reasonable request.

## Abbreviations

ALT: Alanine Aminotransferase
COVID-19: Coronavirus Disease 2019
HFNC: High-Flow Nasal Cannula
IQR: Interquartile Range
IgM: Immunoglobulin M
IgG: Immunoglobulin G
LOS: Length of Stay
pARDS: Pediatric Acute Respiratory Distress Syndrome
PCR: Polymerase Chain Reaction
PICU: Pediatric Intensive Care Unit
SpO₂: Peripheral Oxygen Saturation
SPSS: Statistical Package for the Social Sciences
WHO: World Health Organization
