# Supplementary material for: Clinical characteristics and outcomes of children with measles requiring pediatric intensive care: a multicenter study in Istanbul
Source: Eur J Pediatr. 2026 May 7;185(6):356. doi: 10.1007/s00431-026-07033-y (PMC13149679; doi:10.1007/s00431-026-07033-y)
Supplement: Supplementary file 1 — Supplementary file1 (DOCX 14 KB) [file 431_2026_7033_MOESM1_ESM.docx]

**Supplementary Table S1. Participating Pediatric Intensive Care Units**

The study was conducted across ten pediatric intensive care units (PICUs) in İstanbul, Türkiye. The participating centers were:

1. Department of Pediatric Intensive Care Unit, University of Health Sciences Kanuni Sultan Süleyman Training and Research Hospital, İstanbul, Türkiye
2. Department of Pediatric Intensive Care Unit, University of Health Sciences Bakırköy Dr. Sadi Konuk Training and Research Hospital, İstanbul, Türkiye
3. Department of Pediatric Intensive Care Unit, University of Health Sciences Sancaktepe Training and Research Hospital, İstanbul, Türkiye
4. Department of Pediatric Intensive Care Unit, İstanbul Medipol University, İstanbul, Türkiye
5. Department of Pediatric Intensive Care Unit, University of Health Sciences Haseki Training and Research Hospital, İstanbul, Türkiye
6. Department of Pediatric Intensive Care Unit, University of Health Sciences Başakşehir Çam and Sakura City Hospital, İstanbul, Türkiye
7. Department of Pediatric Intensive Care Unit, İstanbul University, Faculty of Medicine, İstanbul, Türkiye
8. Department of Pediatric Intensive Care Unit, University of Health Sciences Kartal Dr. Lütfi Kırdar City Hospital, İstanbul, Türkiye
9. Department of Pediatric Intensive Care Unit, İstanbul Medeniyet University, Göztepe Prof. Dr. Süleyman Yalçın City Hospital, İstanbul, Türkiye
10. Department of Pediatric Intensive Care Unit, University of Health Sciences Bağcılar Training and Research Hospital, İstanbul, Türkiye
